# De novo variation in *ARID1B* gene causes Coffin-Siris syndrome 1 in a Chinese family with excessive early-onset high myopia

**DOI:** 10.1186/s12920-024-01904-9

**Published:** 2024-05-24

**Authors:** Xiaoyu Huang, Huiping Li, Shangying Yang, Meijiao Ma, Yuanyuan Lian, Xueli Wu, Xiaolong Qi, Xuhui Wang, Weining Rong, Xunlun Sheng

**Affiliations:** 1 Ningxia Eye Hospital, People’s Hospital of Ningxia Hui Autonomous Region, Third Clinical Medical College of Ningxia Medical University, Yinchuan, China; 2 Chaoju Eye Hospital, Hohhot, China; 3 Gansu Aier Ophthalmology and Optometry Hospital, Lanzhou, China

**Keywords:** Coffin-Siris syndrome 1, *ARID1B* gene, Early-onset high myopia, Genotype, Phenotype

## Abstract

Coffin-Siris syndrome (CSS) is a rare autosomal dominant inheritance disorder characterized by distinctive facial features, hypoplasia of the distal phalanx or nail of the fifth and additional digits, developmental or cognitive delay of varying degree, hypotonia, hirsutism/hypertrichosis, sparse scalp hair and varying kind of congenital anomalies. CSS can easily be misdiagnosed as other syndromes or disorders with a similar clinical picture because of their genetic and phenotypic heterogeneity. We describde the genotype-phenotype correlation of one patient from a healthy Chinese family with a novel genotype underlying CSS, who was first diagnosed in the ophthalmology department as early-onset high myopia (eoHM). Comprehensive ophthalmic tests as well as other systemic examinations were performed on participants to confirm the phenotype. The genotype was identified using whole exome sequencing, and further verified the results among other family members by Sanger sequencing. Real-time quantitative PCR (RT-qPCR) technology was used to detect the relative mRNA expression levels of candidate genes between proband and normal family members. The pathogenicity of the identified variant was determined by The American College of Medical Genetics and Genomics (ACMG) guidelines. STRING protein-protein interactions (PPIs) network analysis was used to detect the interaction of candidate gene-related proteins with high myopia gene-related proteins. The patient had excessive eoHM, cone-rod dystrophy, coarse face, excessive hair growth on the face, sparse scalp hair, developmental delay, intellectual disability, moderate hearing loss, dental hypoplasia, patent foramen ovale, chronic non-atrophic gastritis, bilateral renal cysts, cisterna magna, and emotional outbursts with aggression. The genetic assessment revealed that the patient carries a de novo heterozygous frameshift insertion variant in the *ARID1B* c.3981dup (p.Glu1328ArgfsTer5), which are strongly associated with the typical clinical features of CSS patients. The test results of RT-qPCR showed that mRNA expression of the *ARID1B* gene in the proband was approximately 30% lower than that of the normal control in the family, suggesting that the variant had an impact on the gene function at the level of mRNA expression. The variant was pathogenic as assessed by ACMG guidelines. Analysis of protein interactions in the STRING online database revealed that the ARID1A protein interacts with the high myopia gene-related proteins FGFR3, ASXL1, ERBB3, and SOX4, whereas the ARID1A protein antagonizes the ARID1B protein. Therefore, in this paper, we are the first to report a de novo heterozygous frameshift insertion variant in the *ARID1B* gene causing CSS with excessive eoHM. Our study extends the genotypic and phenotypic spectrums for *ARID1B*-CSS and supplies evidence of significant association of eoHM with variant in *ARID1B* gene. As CSS has high genetic and phenotypic heterogeneity, our findings highlight the importance of molecular genetic testing and an interdisciplinary clinical diagnostic workup to avoid misdiagnosis as some disorders with similar manifestations of CSS.

## Introduction

Coffin-Siris syndrome (CSS) is an autosomal dominant disorder with genetic heterogeneity, which was first described and reported by Grange S. Coffin and Evelyn Siris in 1970 [1]. It is characterized by developmental delay, intellectual disability, coarse facial features, aplasia or hypoplasia of the distal phalanx or nail of the fifth and additional digits, hypotonia, hypertrichosis, and physical multiorgan dysplasia [2]. *ARID1B*-Related Disorder (*ARID1B*-RD) is caused by heterozygous pathogenic variants of *ARID1B*, including classic CSS and intellectual disability with or without nonspecific dysmorphic features. The incidence is estimated statistically to be 1:10,000 to 1:100,000 [3].

CSS genotype-phenotype correlations are unclear yet. The typical coarse facial features include thick eyebrows, long eyelashes, low-set posteriorly rotated ears, depressed nasal bridge, wide nasal tip, thin upper lip, and thick lower lip [2]. The physical features include retardation of postnatal growth, feeding difficulties, mental retardation, speech & language developmental disorders, fifth finger/toe hypoplasia, hypotonia, joint laxity, scoliosis, lumbar disc stenosis, congenital heart defect, recurrent upper respiratory infections [2], intestinal obstructions, gastric ulcer, inguinal hernia, cryptorchidism, subcutaneous hemangioma [4], melanocytic nevus, vitiligo [5], agenesis of the corpus callosum, arachnoid cyst, hearing loss, visual loss, dental hypoplasia, epilepsy, autism, and aggression [2]. Ocular symptoms of CSS may also manifest as ptosis, microphthalmos, anterior segment dysgenesis, congenital or secondary glaucoma, cataracts, astigmatism, strabismus, hyperopia, amblyopia, nystagmus [6], blue sclera [4], night blindness [2], vernal keratoconjunctivitis [7], color vision abnormalities, and retinal dystrophy [8]. Van der Sluijs PJ et al. reported that approximately 48.6% of patients had visual impairment in a study consisting of 143 patients with the *ARID1B* variant, of which myopia accounted for 27.5%, ranging in severity from − 24.00D to -1.00D (median − 6.35D) [9].

CSS is caused by mutations in the gene encoding the human BRG1-associated factor (BAF) chromatin-remodeling complex (also known as the SWI/SNF-A complex) [10]. BAF acts as an epigenetic modifier by altering chromatin structure, thereby facilitating access to DNA by transcription factors [10]. A total of 12 types of CSS caused by 12 genetic variants have been reported so far, including *ARID1B* (MIM#614,556; CSS1 MIM#135,900), *ARID1A* (MIM#603,024; CSS2 MIM#614,607), *SMARCB1* (MIM#601,607; CSS3 MIM#614,608), *SMARCA4* (MIM#603,254; CSS4 MIM#614,609), *SMARCE1* (MIM#603,111; CSS5 MIM#616,938), *ARID2* (MIM#609,539; CSS6 MIM#617,808), *DPF2* (MIM# 601,671; CSS7 MIM#618,027), *SMARCC2* (MIM#601,734; CSS8 MIM#618,362), *SOX11* (MIM#600,898; CSS9 MIM#615,866), *SOX4* (MIM#184,430; CSS10 MIM#618,506), *SMARCD1* (MIM#601,735; CSS11 MIM#618,779), *BICRA* (MIM#605,690; CSS12 MIM#619,325) (https://www.omim.org/). Of these, *ARID1B* is by far the most commonly variant gene (51–75%) [9]. *ARID1B* is located on chromosome 6q25.3 and contains 20 exons encoding an AT-rich interactive domain (ARID) protein containing 2249 amino acids (NP_065783.3), a subunit of the Brahma-associated factor (BAF) complex [3]. *ARID1B* is mainly expressed in differentiated cell types but is also thought to be involved in early brain development [11]. The ARID1B protein functions antagonistically with the ARID1A protein, which is also rich in AT structural domains, and both have important roles in cell cycle regulation [10]. Almost all *ARID1B* gene variants are non-functional, including nonsense variants, frameshift variants, splicing site variants, and single-exon/multiple-exon/whole-gene deletions [3, 10].

Early-onset high myopia (eoHM), defined as a high myopia onset before school age, is considered to be predominantly determined by genetic factors with minimal environmental effects [12]. Variations in at least 17 genes have been reported to be responsible for eoHM, including *SCO2* (MIM#604,272), *P4HA2* (MIM#600,608), *LRPAP1* (MIM#104,225), *ZNF644* (MIM#614,159), *SLC39A5* (MIM#608,730), *LEPREL* (MIM#610,341), *ARR3* (MIM#301,770), *OPN1LW* (MIM#300,822), *BSG* (MIM#109,480), *CPSF1* (MIM#606,027), *LOXL3* (MIM#607,163), *NDUFAF7* (MIM#615,898), *TNFRSF21* (MIM#605,732) [12], *CCDC111* (MIM#615,421), *XYLT1* (MIM#608,124), *DZIP1* (MIM#608,671), and *CTSH* (MIM#116,820) [13]. However, variations in these genes may only explain a small part of patients with eoHM and the genetic defects for most patients with eoHM are yet unknown and may be caused by variants in unknown novel genes. Some studies showed that eoHM may occur associated with other ocular or systemic diseases and so associated with the responsible genes, such as *LPR2* causing Donnai-Barrow syndrome with eoHM [14] and *EP300* causing Rubinstein-Taybi syndrome 2 with eoHM [15].

## Materials and methods

### General data

The pedigree study was used to collect one CSS 1 Chinese family of Han nationality who presented to the Ningxia Eye Hospital in July 2021 for blurred vision since childhood. The family consists of 4 individuals in 2 generations, including 1 patient. To ask in detail about the current medical history, past history, birth history, family history, parents’ marriage and childbearing history of the patient, and the pedigree chart was drawn. General physical examination and eye examination were performed on the proband and family members, and photographs were taken to record the proband’s facial, hand, and foot features. The study project was approved by the Ethics Committee of the People Hospital of Ningxia Hui Autonomous Region [Approval No. 2022-LL-022] and in strict compliance with the Declaration of Helsinki. Written informed consent was received from each participant or his/her legal guardians before participation.

### Methods

#### Ophthalmic examination

The anterior segment was examined by the slit lamp microscope (Topcon, Japan). The intraocular pressure (IOP) of the subjects was measured by a non-contact tonometer (NIDEK NT-510, Japan). The best corrected visual acuity (BCVA) was recorded by the snellen visual acuity chart and optometrist (VT-10, Topcon, Japan & ARK-1, Japan). The axial length (AL) was measured by an IOL-Master optical biometer (IOL Master 700, Carl Zeiss Meditec AG). The fundus of the subjects was examined by color fundus photography (TRC-NW300, Topcon, Japan), ultra-wide angle fundus photography (Panoramic Ophthalmoscope 200, UK), optical coherence tomography (OCT, Spectralis OCT, Heidelberg Engineering GmbH) and electroretinography (ERG, LCK TECHNOLOGIES (RETeval), USA).

### DNA extraction, library construction, and whole exome sequencing

2–3 mL of peripheral venous blood of the proband, his parents, and sister were collected into the EDTA-K2 anticoagulant tubes, thoroughly mixed upside down, and stored in the refrigerator at -80℃ ready for use. DNA was rapidly extracted according to the instructions of the DNA Extraction Kit (TIANamp Blood DNA Kit, #DP348-03, Beijing Tiangen Co. Ltd.), and DNA concentration and purity were examined using NanoDrop 2000 UV Spectrophotometer (Thermo Fisher, Nanodrop, U.S.A.), and stored in a refrigerator at -20℃. The whole exome library was constructed using customized probes from Shanghai Weihansi Biomedical Technology Co., Ltd. and synthesized by TWIST followed by whole exome library capture. WES was performed on the proband samples with an average sequencing depth of 150X. For analysis of sequencing data, the reads that did not meet the quality control requirements in the original sequencing data were removed first, and then BWA (Burrows-Wheeler Aligner) software was used to compare with the reference sequence of the hg19 version of the human genome provided by UCSC (https://genome.ucsc.edu/). Finally, GATK v3.70 (Genome Analysis Toolkit) was used to identify the variation. Databases such as gnomAD_exome, ExAC, and 1000genomes were used to test the frequency of target variants across races, which needed to be excluded when the frequency of the tested variants was higher than 2% of the normal population.

### Bioinformatics analysis and Sanger sequencing

Database tools such as HGMD (human gene mutation database) and dbSNP (https://www.ncbi.nlm.nih.gov/snp/) were utilized to search the target variant site, and check in HGMD to see if it is a reported pathogenic variant and see if it has been included. In the case of new or de novo variants that had not been reported, the scoring of the genetic variant as well as its pathogenicity were further assessed according to Standards and Guidelines for Interpretation of Sequence Variants issued by the American College of Medical Genetics and Genomics (ACMG) in 2015, categorizing them as pathogenic, likely pathogenic, uncertain significance, likely benign, and benign [16]. MAF<0.005 was used as the criteria to exclude benign variants by reference to the databases for East Asian populations Allele frequencies available with Exome Aggregation Consortium (http://exac.broadinstitute.org/) and 1000 Genomes Project (http://browser.1000genomes.org). Nonsense variants, frameshift variants, and variants with experimental evidence of loss of protein function were classified as pathogenic variants. MEGA and ClustalX software were used to analyze the evolutionary conservation of target amino acids of *ARID1B* protein. Co-segregation analysis was performed among members of the patient’s family using Sanger sequencing.

### Structural analysis

Protein modeling of the wild-type and mutant ARID1B were constructed with AlphaFold (https://alphafold.ebi.ac.uk), evaluated by SAVES6.0 (https://saves.mbi.ucla.edu/) and displayed with PyMol software (https://pymol.org/2/).

### Protein-protein interaction (PPI) network analysis

Access the STRING website (https://cn.string-db.org/), click on “Pathway/Process/Disease” in the left column, enter the disease name “high myopia” in the “Search term” on the right, and select “Homo sapiens” in " Organisms”, and click “SEARCH” to get the protein relationship diagram associated with high myopia, and click “protein node degrees” under “Exports”. Click " protein node degrees: download " under “Exports” to get the text of myopia-related proteins. Return to the main page, click on “Multiple proteins” in the left column, and enter the known CSS-related protein (ARID1B ARID1A SMARCA4 SMARCB1 SMARCE1 ARID2 DPF2 SMARCC2 SOX11 SOX4 SMARCD1 BICRA) and the downloaded text of high myopia-related protein on the right. Select “Homo sapiens” in “Organisms”, click “SEARCH”, and click “CONTINUE” to get the relationship diagram between CSS-related proteins and high myopia-related proteins. Click “as a high-resolution bitmap: download” under “Exports” to get the overall result picture. Screen the protein text directly related to CSS-related proteins and high myopia related proteins, and repeat the above steps of “Multiple proteins” to simplify the results.

### Relative mRNA expression of *ARID1B* gene

The relative mRNA expression of *ARID1B* gene was examined in samples from proband and his mother (normal control). RNA was extracted from blood samples using the E.Z.N.A.™PX RNA Kit (Omega#R1057-02) and reverse transcription was performed using the kit HifairTM II 1st Strand cDNA Synthesis Kit (gDNA digester plus) (Yeason Biotech, 11121ES50). A pair of specific primers were designed on Exon3 of the *ARID1B* gene (forward:5’ GCAAGGTGTGAGTGGTTACTG3’ and reverse:5’ GGACTGGGGACGGCAGATACT3’), the RT-qPCR reaction system was prepared according to the NovoStart® SYBR qPCR SuperMix Plus kit. The 10 µL system contained 5 µL of 2×NovoStart® SYBR qPCR SuperMix Plus, 0.5 µL of each forward and reverse primer, and 4 µL of cDNA. A program of initial denaturation (95°C for 1 min) and 40 cycles of amplification (95°C for 20 s, 60°C for 20 s) was performed on a Roche Light Cycler II 480 real-time fluorescent quantitative PCR instrument with 3 replicates set for each reaction. The 2-ΔΔCt method was used to calculate the relative mRNA expression of EP300 gene, and the GAPDH gene was used as the internal reference gene (forward:5’ AAATCAAGTGGGGGCGATGCT3’ and reverse:5’ GATGACCCTTTTGGGCTCCCC3’).

## Results

### Clinical features of pedigree

**Fig. 1:**
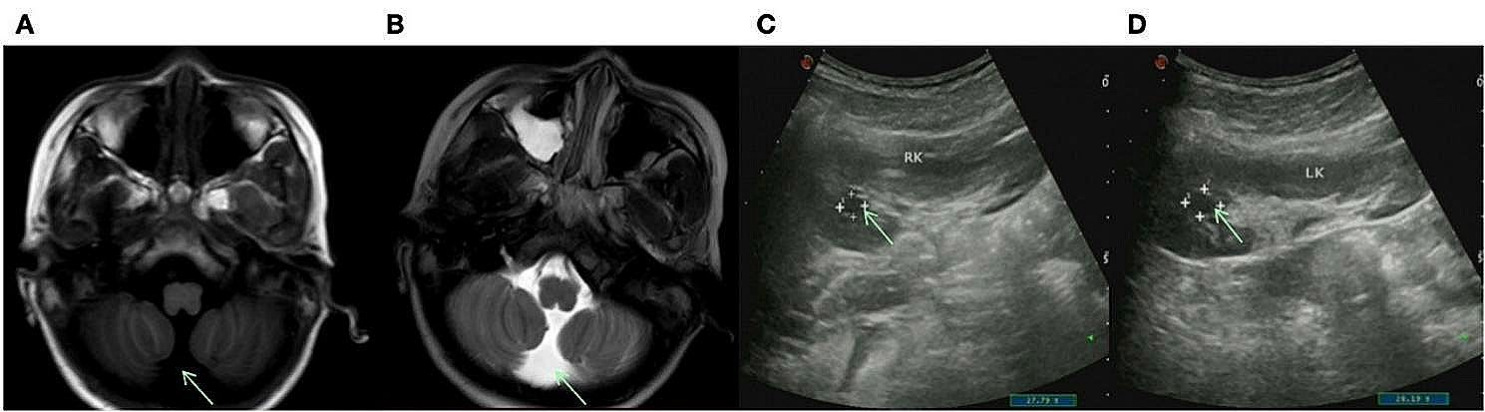
Magnetic resonance imaging (MRI) of the brain and color ultrasound of the kidney in our proband. **(AB)** MRI showed low signal at T1WI and high signal at T2WI, suggesting cisterna magna, a congenital anatomical variant. **(CD)** Renal color ultrasonography revealed a 0.7 × 0.8 cm cystic dark area in the right kidney and a 1.1 × 0.8 cm cystic dark area in the left kidney, suggesting bilateral renal cysts

**Fig. 2:**
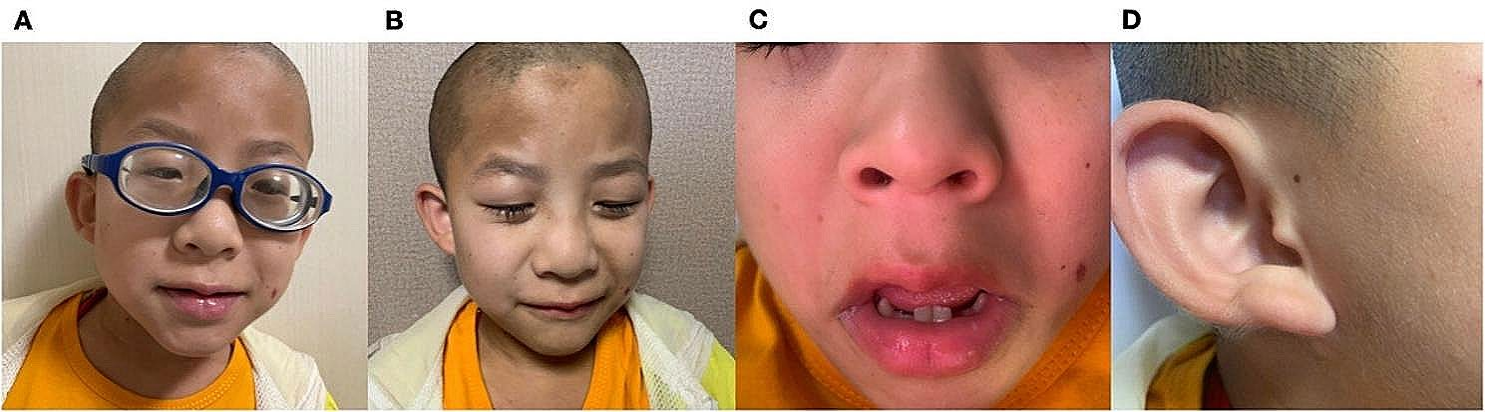
Facial features of the proband. **(A)** Sparse scalp hair and high myopia wearing frame glasses of thick lenses. **(B)** Coarseness face: thick eyebrows, long eyelashes, broad nasal bridge with broad nasal tip. **(C)** The wide mouth with thick, everted lower lip and thin upper lip and delayed dentition associated with irregular teeth. **(D)** Excessive pale yellow fine hairs growth on the face and ears and low-set posteriorly rotated ears

Proband, male, unrelated parents, full-term normal delivery, birth weight 2.3 kg (< 2.5 kg), breast-fed and formula-fed since birth, and no feeding difficulties. Diagnosed with autism at 2 years old, walked at 3 years old, underwent entropion correction at 4 years old due to bilateral entropion and trichiasis, was found to have high myopia in both eyes, and was found to have moderately impaired hearing in both ears as examined by brain-stem evoked potential and homeostatic auditory system at 6 years old, able to repeat the sounds at 7 years old, able to pronounce the sounds fluently at the age of 9 years old, and found to have cisterna magna, patent foramen ovale, chronic non-atrophic gastritis and bilateral renal cysts at 10 years old (Fig. 1). Now 12 years old, his height/weight is 135.5 cm/28.2 kg (BMI:15.4 kg/m^2^ < 18 kg/m^2^), head circumference is 52 cm (normal size). Facial features (Fig. 2): Coarse face, thick eyebrows, long eyelashes, low-set posteriorly rotated ears, low nasal bridge, broad nasal tip, thin upper lip & thick lower lip, and excessive hair growth on the face; Other features: Dental hypoplasia, intellectual disability, inability to write numbers, and emotional outbursts with aggression. The mother and sister had no significant clinical phenotypes.

**Fig. 3:**
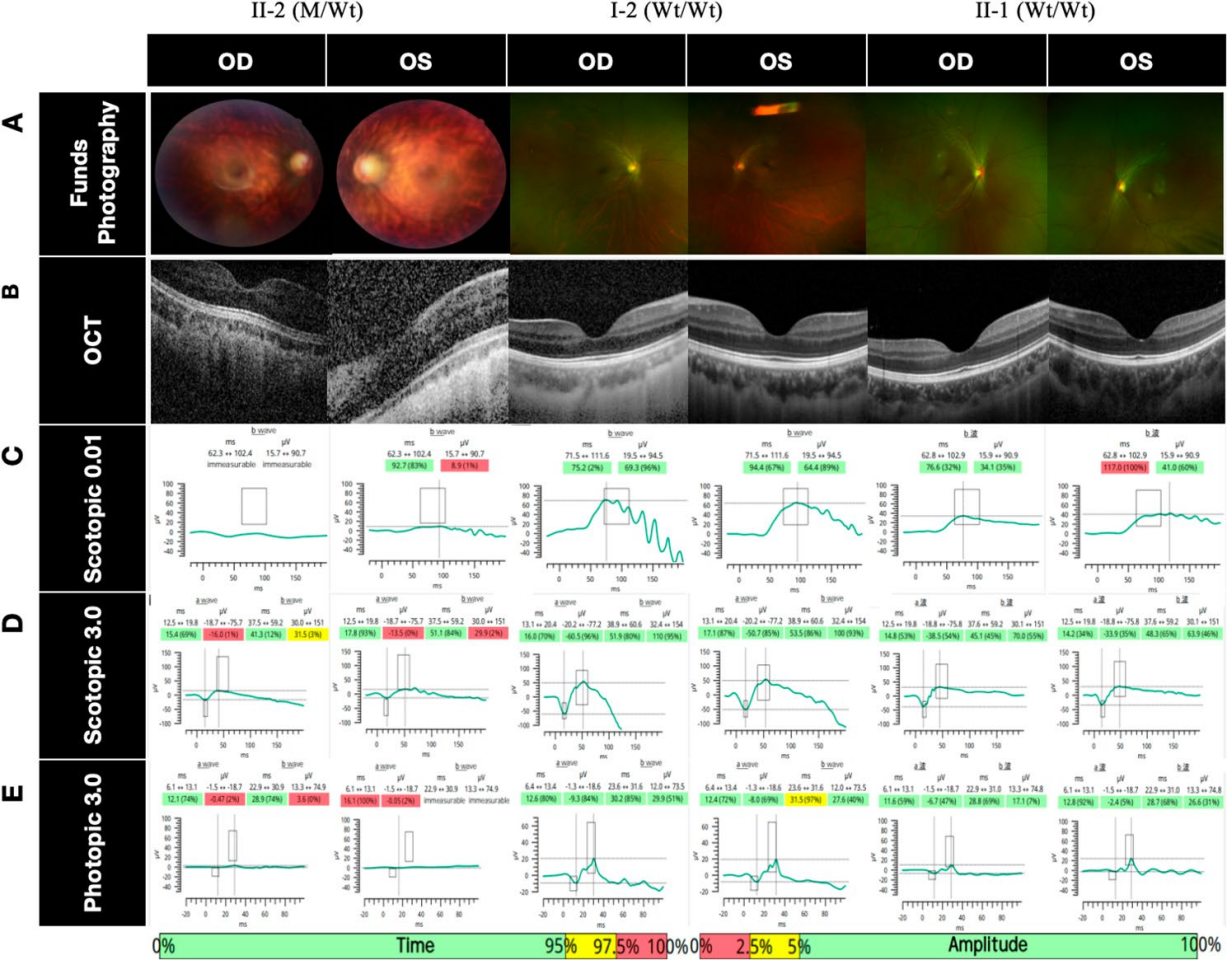
Ophthalmologic imaging of the proband, his mother, and his sister. **(A)** Fundus photography: II-2 showed leopard fundus changes in both eyes; I-2 and II-1 showed no abnormalities. **(B)** Macular OCT: II-2, I-2, and II-1 showed no abnormalities. **(CDE)** ERG: Referring to the normal reference range of waveform time and amplitude shown below, II-2 has decreased amplitude of scotopic adaptation and photopic adaptation a- and b-waves, and no abnormality is seen in I-2 and II-1, suggesting dystrophy of the cone and rod cells in II-2

The proband had excessive high myopia, astigmatism, and amblyopia in both eyes. Mydriatic optometry with Compound Tropicamide and the best corrected visual acuity: 0.2 (-16.00DS/-8.25DCx5°) in the right eye and 0.1 (-24.00DS/-8.25DCx120°) in the left eye. The axial length was measured by the IOL-Master optical biometric instrument: 30.00 mm in the right eye and 31.97 mm in the left eye (Table 1). Slit-lamp microscopy of the anterior segment revealed opacification of the posterior capsula in both eyes. Color fundus photography showed leopard fundus changes in both eyes. Macular OCT showed no abnormality. ERG suggests cone-rod cell dystrophy. No significant abnormalities were found in the mother and sister’s ophthalmologic examination (Fig. 3). The best corrected visual acuity and color fundus photography in this article are the results of the examination at the age of 10 years (2021) due to the inability of the proband to fully cooperate with this examination, and the current intraocular pressure was measured with finger palpation to be normal.

### Genetic test results

**Fig. 4:**
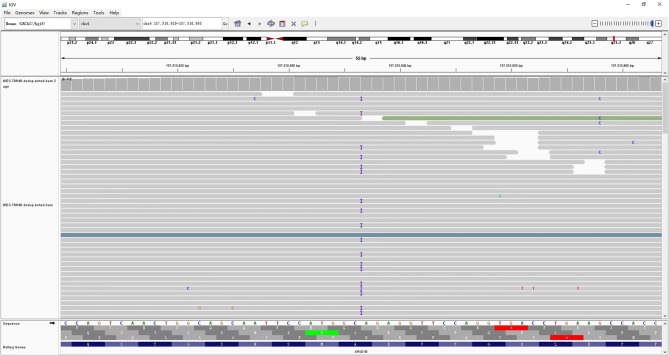
Integrative Genomics Viewer: the WES analysis of the mutation

**Fig. 5:**
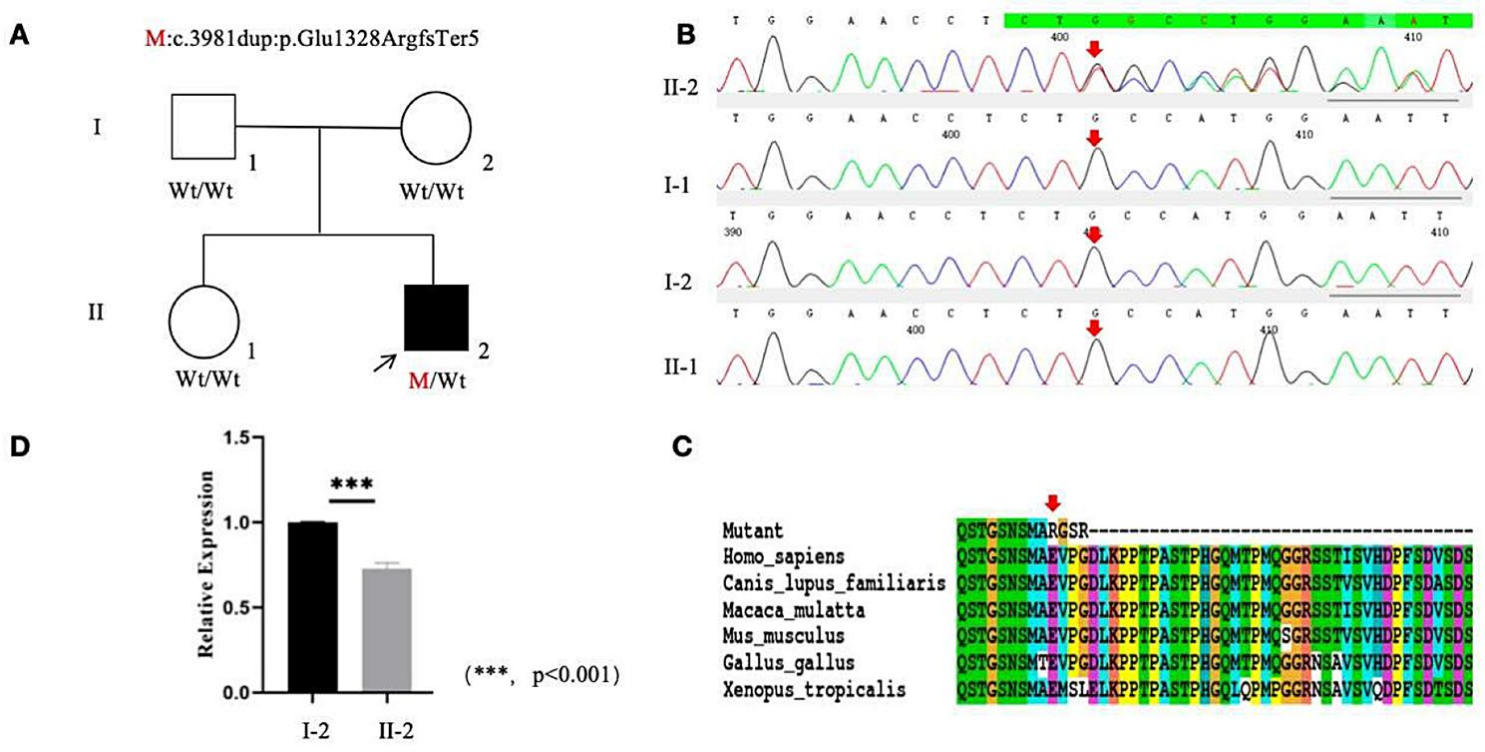
Sequence analysis and identification of the novel mutation of *ARID1B* in the affected family with autosomal-dominant CSS. **(A)** Pedigree. The filled black symbol represents the affected member and the arrow denotes the proband. **(B)** Sequence chromatograms of identified mutation. **(C)** The homology of amino acid sequences between human *ARID1B* and other species. **(D)** Relative gene expression levels of *ARID1B* in the affected family with autosomal-dominant CSS: showing relative gene expression levels of *ARID1B* of II:2(0.71) is lower than I:2(1.0), *p* < 0.001

A heterozygous frameshift insertion variant M:*ARID1B*: NM_001374828.1:exon14:c.3981dup (p.Glu1328ArgfsTer5) detected in the *ARID1B* gene of the proband by whole exome sequencing was located in chromosome 6q25.3 (Fig. 4). Sanger sequencing confirmed that neither the parents of the proband nor his sister carried such a variant. This variant resulted in the repetition of base A at site 3981 of exon 14 of the sequence, and frameshift and nonsense variants at site 1328 of the corresponding protein sequence, where glutamate was changed to arginine, leading to premature termination of protein translation and affecting the normal function of the protein. According to Standards and Guidelines for Interpretation of Sequence Variants, the nonsense variant was Pathogenic Very Strong (PVS1). The phenotypically normal parents and sister did not carry such a variant, suggesting that the variant was de novo and classified as Pathogenic Strong (PS2). The variant was not found in the normal population database and thus classified as Pathogenic Moderate (PM2). And co-segregation of the variant in the pedigrees as Pathogenic Supporting (PP1). Amino acid conservation analysis identified glutamate at site 1328 of the translated amino acid sequence of the *ARID1B* gene, which was highly conserved in Homo sapiens, Mus musculus, Canis lupus familiaris, Xenopus tropicalis, Macaca mulatta, Gallus gallus as Pathogenic Supporting (PP3). Such variant was classified as pathogenic (PVS1 + PS2 + PM2 + PP1 + PP3) as assessed by the standards and guidelines for the interpretation of sequence variants (Fig. 5).

**Fig. 6:**
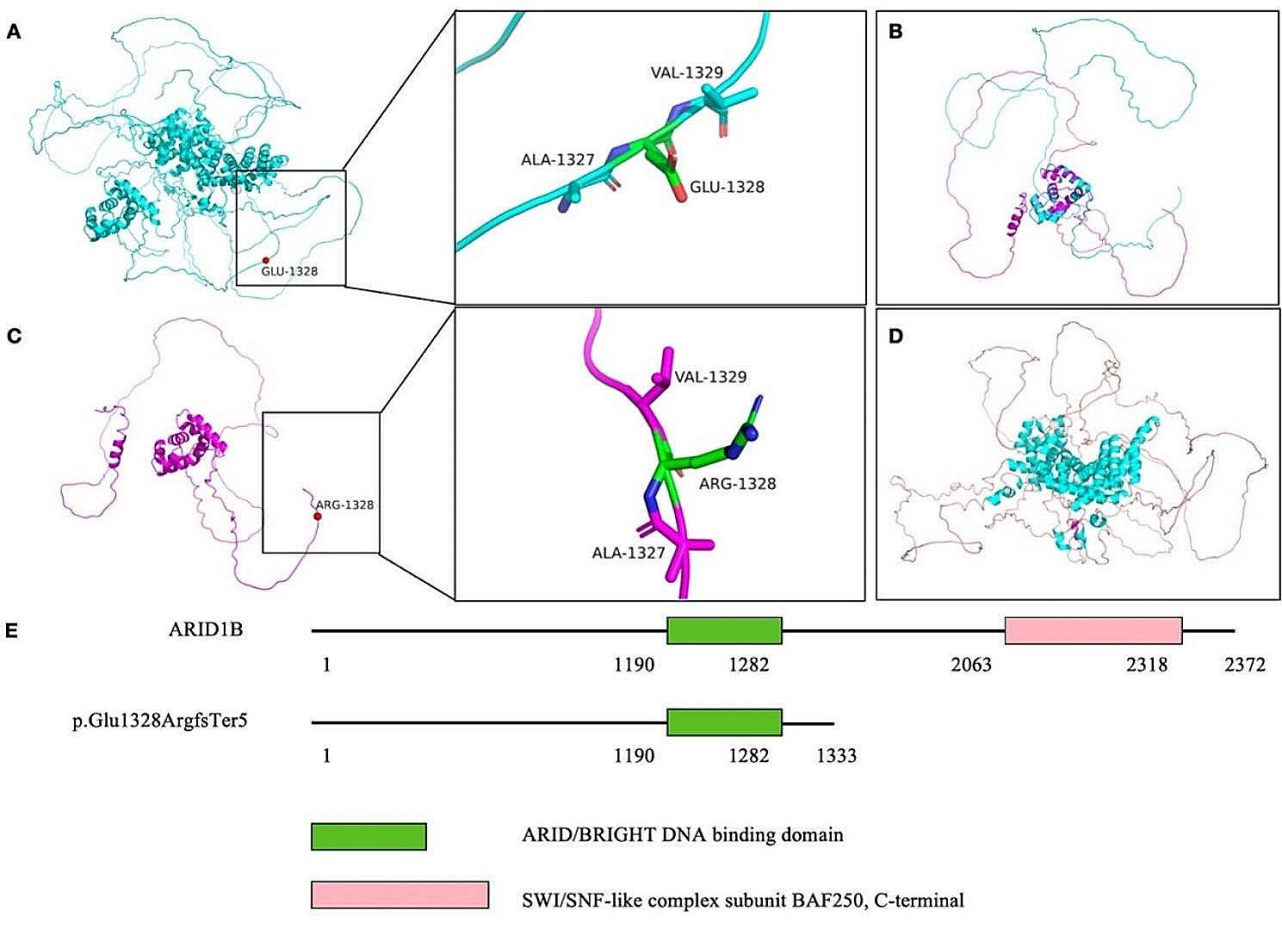
Structural analysis of the wild-type and mutant ARID1B protein. (**AC**) The 1328 position of the wild-type ARID1B protein is negatively charged glutamate, which is located in the loop domain. The 1328 position of the mutant ARID1B protein(p.Glu1328ArgfsTer5) is positively charged arginine. (**B**) The superposition of the first 1000–1333 amino acid structure of the wild-type and mutant ARID1B protein results in the termination of translation due to mutation, and the mutant has a helical structure at segments 1015–1024, while the wild-type does not. (**D**) Translation terminates at 1333 position and the structures that cause protein deletion which consist mainly of 27 alpha helices, 2 beta folds, and numerous loop structures. (**E**) The SWI/SNF-like complex subunit BAF250 and C-terminal functional domain of ARIDIB mutant protein are missing

Structural analysis revealed that the wild-type ARID1B protein has a negatively charged glutamic acid at position 1328 and the mutant ARID1B protein changes to positively charged arginine at position 1328. The mutant ARID1B protein has a helical structure in the segment 1015–1024, while the wild-type does not. Translation is terminated at position 1333, resulting in the absence of the protein’s structure, which consists mainly of 27 α-helices, 2 β-folds and numerous loop structures. All above changes may influence protein function. Domain mapping suggested that the mutation(p.Glu1328ArgfsTer5) results in deletion of the SWI/SNF-like complex subunit BAF250, C-terminal functional domain(Fig. 6).

**Fig. 7:**
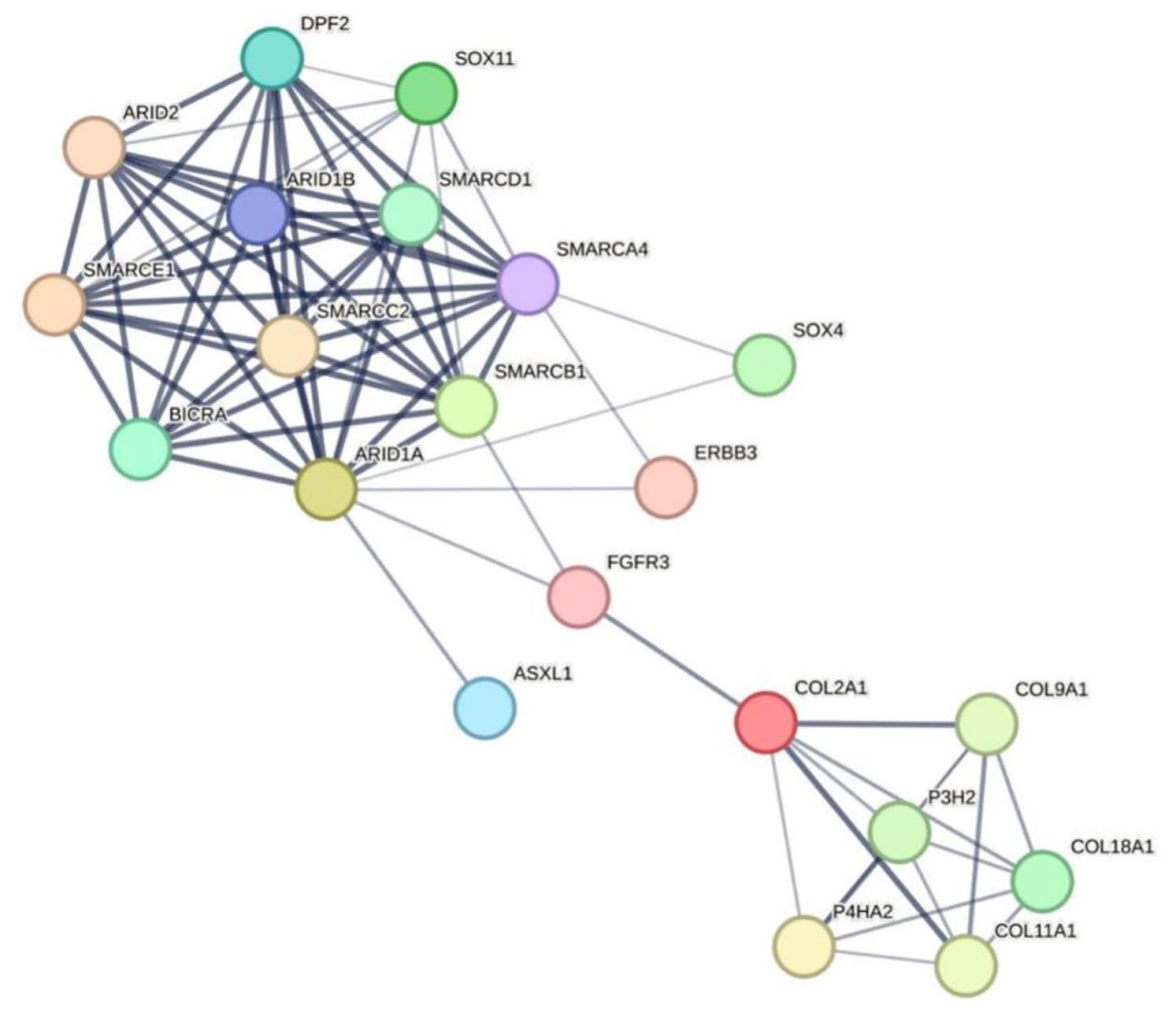
Protein interactions diagram. A simplified part of high myopia-related proteins with CSS-related proteins in the STRING online database. The high myopia-related proteins (ASXL1, FGFR3, ERBB3, SOX4) interacted with ARIDIA proteins, and ARIDIA proteins interacted with ARID1B proteins. COL2A1 protein affects FGFR3 protein which in turn affects ARIDIA protein, suggesting that proteins with a strong association with high myopia may contribute to the clinical phenotype of high myopia in CSS patients through protein interactions

Proteins with a strong association with high myopia (ADAMTS10 ADAMTSL4 ARR3 ASXL1 ATP6V0A2 ATP6V1A ATP6V1B2 ATP6V1E1 CNGB3 COL11A1 COL18A1 COL2A1 COL9A1 CPSF1 CRIPT CYP4V2 DAG1 EFL1 ELOVL4 ERBB3 FBN1 FGFR3 GZF1 HERC1 IRX5 KAT5 LRP2 LRPAP1 LTBP2 MYOC NYX P3H2 P4HA2 PRDM5 RAB28 RIN2 SLITRK6 SMS TBC1D24 TGFBI ZNF469 ZNF644) were obtained using STRING online database and its interaction with CSS-related proteins were analyzed. It was found that high myopic associated proteins (ASXL1 FGFR3 ERBB3 SOX4) can affect ARID1B protein by affecting ARIDIA protein, and COL2A1 protein can also affect ARIDIA protein by affecting FGFR3 protein, resulting in the clinical phenotype of patients with high myopia (Fig. 7).

RT-qPCR method was used to detect the expression level of *ARID1B* gene transcription - relative mRNA expression level in proband and a normal family member (control group). The results showed that the relative mRNA expression level of *ARID1B* gene in the proband sample (0.71) was lower than that in the control group (1.0), *P* < 0.001, which was about 30% lower as compared with the control group. This suggests that most mutated transcripts are eliminated before translation due to nonsense-mediated mRNA degradation (Fig. 5).

Based on the clinical phenotype (typical facial features, developmental delay, hirsutism, autism, congenital heart disease, and high myopia) and 1 heterozygous frameshift insertion variant (c.3981dup) on the *ARID1B* gene, the patient was diagnosed with CSS 1.

## Discussion

CSS is a rare autosomal dominant genetic disorder that often involves multiple organs and systems throughout the body, and the genetic and clinical phenotypes are highly heterogeneous. Therefore, CSS cannot be completely diagnosed and typed by clinical manifestations alone, and genetic detection of pathogenic genes such as *ARID1B* is the gold standard for CSS 1 diagnosis.

In this study, WES was utilized to identify 1 heterozygous frameshift insertion variant M: *ARID1B*: NM_001374828.1:exon14:c.3981dup (p.Glu1328ArgfsTer5). Bioinformatics analysis demonstrated the pathogenicity of the above variant, confirming the diagnosis of CSS 1. *ARID1B*, by far the most common variant gene (51–75%), is located on chromosome 6q25.3 and contains 20 exons, and its variations result in nonsense variants. Nonsense-mediated mRNA decay (NMD) which refers to the presence of a premature termination codon (PTC) on an mRNA under pathological or normal physiological conditions, thus leading to mRNA decay, is a widespread mechanism for mRNA quality monitoring [17]. In pathological situations, genetic variants, mainly nonsense and frameshift ones, can generate PTCs that cause disease by triggering NMD decay of mRNA leading to decreased expression of target proteins [18]. Approximately 1/3 of human genetic disorders of known etiology are caused by PTCs generated by genetic variants [19]. In most cases, the NMD pathway is triggered only when the PTC and its downstream neighboring exon-exon junction (EEJ) are greater than or equal to 50 ?∼ 55 nucleotides [20]. The variant in this study is located at exon 14, 63 nucleotides from EEJ, which triggers the NMD pathway to reduce mRNA expression. Previously Grochowski CM [21] et al. and Halgren C [22] et al. reported an approximately 30% reduction in the expression level of the relative mRNA expression of the *ARID1B* gene in blood samples from CSS1 patients caused by the *ARID1B* variant, respectively, and our patients were also tested and found to have an approximately 30% reduction in the relative mRNA expression of the *ARID1B* gene in comparison with the control group. Due to the mechanism of nonsense-mediated mRNA decay, most of the mutated transcripts are eliminated before translation, and transcripts carrying such PTCs have significantly lower levels of mRNA transcripts than the wild-type proteins, and the resulting disease symptoms are usually milder.

In this study, the clinical manifestations of the proband were consistent with previous reports in the literature, including coarse facial features, growth/intelligence/language & speech delays, hypotonia, hypertrichosis, abnormal organ development, vision loss, and hearing loss. However entropion and trichiasis have not been reported in previous cases. Among them, visual loss arising from early-onset high myopia was the prominent ocular manifestation, previously Pranckėnienė L et al [23] reported a case of a 13-year-old female CSS1 patient whose refractive error indicated − 23.00 DS in the right eye and − 22.00 DS in the left eye, while other case reports did not provide specific refractive error or only reported myopia. Complex cellular events, such as cell cycle control and chromosome separation, require interactions between many gene products, and a single gene may interact with an average of 10–15 genes, so that the gene network of the entire cell will be huge and highly complex [24]. We predicted that ARID1A protein interacts with high myopia gene-related proteins FGFR3, ASXL1, ERBB3, and SOX4, and ARID1A protein antagonizes ARID1B protein, which may affect proteins with a strong association with high myopia by affecting ARID1A protein, leading to the clinical phenotype of high myopia in patients. Take the COL2A1-FGFR3-ARIDIA-ARID1B interaction as an example. The *COL2A1* gene encodes the α1 chain of type II collagen, which is primarily expressed in the vitreous body and cartilage. Mutations in the *COL2A1* gene result in Stickler syndrome which often characterized by high myopia [25]. The *FGFR3* gene encodes fibroblast growth factor receptor 3, mainly expressed in cartilage and lens, and mutations in *FGFR3* gene can also lead to clinical manifestations of myopia [26]. Both *COL2A1* and *FGFR3* genes play a role in promoting growth and development, so they may have a synergistic effect. In a study on tumors, it was suggested that *FGFR3* gene had a negative association with mutations in the *ARIDIA* gene [27]. While the ARID1B protein functions antagonistically with the ARID1A protein [10]. Therefore, we predict that mutations in the *ARID1B* gene would result in decreased expression of wild-type protein, relatively enhanced activity of ARIDIA protein, low expression of FGFR3 protein, and low expression of COL2A1 protein leading to clinical phenotypes associated with myopia. High myopia is defined as a refractive error ≤-6.0 D or an ocular axis length > 26 mm [28]. According to the age of onset, high myopia is divided into late-onset high myopia (loHM) which occurs after school age, and early-onset high myopia (eoHM) which occurs before school age [12]. Numerous genetic studies have shown that eoHM is different from loHM. EoHM onset occurs before school age (< 7 years), it is minimally influenced by environmental factors (e.g. close work) but mainly determined by genetic factors [12, 29]. EoHM can be categorized into a simple (nonsyndromic) type that presents only with high myopia, and a syndromic type that combines with other diseases of the eye or abnormalities in other physical systems [30]. EoHM is closely associated with many genetic disorders and is often the primary cause of first diagnosis in some patients and the first clinical feature that comes to the attention of clinicians. Due to the lack of awareness and attention of ophthalmologists to this group of genetic diseases, it is easy to lead to misdiagnosis or missed diagnosis. In this study, the patient was found to be highly myopic at the age of 4 years without further examination and prescription of spectacles. At the age of 9 years, the diagnosis of high myopia was made and was treated with spectacles only without further examination and genetic testing, leading to misdiagnosis. EoHM may be the leading cause of early childhood visits and an important clue for clinicians to identify underlying eye diseases. Therefore, in addition to detailed ocular structural and functional examination of eoHM, genetic screening should be prioritized to identify causative genes, which facilitate early diagnosis, effective intervention, and long-term follow-up evaluation of these diseases.

Currently, there is no specific treatment against CSS but treatment of manifestations with periodic monitoring and symptomatic treatment. It has been commonly used that the standard treatment for refractive error, strabismus, hearing loss, obstructive sleep apnea, congenital heart defects, constipation, gastroesophageal reflux, cryptorchidism, scoliosis, and seizure disorders. Developmental therapies including speech/language and feeding therapy, are recommended for those with developmental delay. Surveillance includes at least annual assessment of developmental progress and educational needs; annual ophthalmology evaluation and assessment for scoliosis (until growth is complete). It is essential for patients to undergo ophthalmologic examinations regularly, in which the assessment of refractive errors and amblyopia and their early correction have a positive impact on child development. The reported cases of CSS 1 included death from cerebrovascular disease at the age of 7 years [1] and survival to the age of 69 years [31]. In a study of 8 fetuses with the prenatal diagnosis of CSS, Qiu-Xia Yu et al [32] found that agenesis of the corpus callosum was the most common ultrasound manifestation, which may be included in the differential diagnosis of CSS for whole exome sequencing.

## Conclusion

Coffin-Siris syndrome (CSS) is a rare complex disorder and can easily be misdiagnosed as other syndromes or disorders with a similar clinical picture because of their genetic and phenotypic heterogeneity. Its clinical manifestations are serious enough to warrant timely diagnosis and management. Here, in this paper, we first report the de novo heterozygous frameshift insertion variant in the *ARID1B* gene that caused CSS in this Chinese family with excessive eoHM. Our study revealed important network modules by PPI network analysis and *ARID1B* gene potentially related to high myopia development, which expanded the list of candidate genes associated with eoHM and implied the patient with eoHM can provide an important clue for genetic screening and further specific clinical examinations to promote accurate assessment and prompt treatment.

**Table 1:** Ophthalmological examination of CSS patient with early-onset high myopia and unaffected members

Subject	Sex	Age	Eye		Refraction	BCAV	IOP(mmHg)	AL (mm)
II-2	M	12	OD	-16.00DS/-8.25DCx5°		0.2	15.0	30.00
			OS	-24.00DS/-8.25DCx120°		0.1	16.0	31.97
I-1	F	33	OD	-3.25DS/-2.50DCx172°		0.9^+^	13.3	24.93
			OS	-3.50DS/-2.50DCx3°		0.9^+^	14.3	24.71
II-1	F	13	OD	-2.25DS/-0.75DCx167°		1.0	15.0	23.97
			OS	-1.00DS/-0.75DCx176°		1.0	13.3	23.51

M: male, F: female, OD: oculus dexter, OS: oculus sinister, BCVA: best corrected visual acuity, IOP: intraocular pressure, AL: axiaI length

## Data Availability

No datasets were generated or analysed during the current study.
